# Resistance of Bamboo Fibers to Gastrointestinal Digestion and Their Nutrient-Dependent Fermentation by Human Gut Microbiota

**DOI:** 10.3390/foods15152740

**Published:** 2026-08-04

**Authors:** Hélène Lefèvre, Khaled Fadhlaoui, Jean-Sébastien Guez, Emmanuelle Lainé, Eric Beyssac

**Affiliations:** 1 UMR 454 MEDIS UCA-INRAE, Université Clermont Auvergne, 63000 Clermont-Ferrand, France; helene.lefevre@uca.fr (H.L.); khaled.fadhlaoui@uca.fr (K.F.); emmanuelle.laine@uca.fr (E.L.); 2 UMR 6023 LMGE CNRS-UCA, Université Clermont Auvergne, 63000 Clermont-Ferrand, France; 3 Institut Pascal, Clermont Auvergne INP, CNRS, Université Clermont Auvergne, 63000 Clermont-Ferrand, France; j-sebastien.guez@uca.fr

**Keywords:** bamboo fiber, gastrointestinal digestion, colonic fermentation, gut microbiota modulation, short-chain fatty acids, functional dietary fiber

## Abstract

Bamboo fibers are increasingly incorporated into food products as sustainable dietary fiber ingredients, yet their gastrointestinal digestion resistance and fermentative behavior by human gut microbiota remain insufficiently characterized. This study combined an INFOGEST-based in vitro digestion protocol with colonic fermentation using fecal microbiota from three healthy donors as an exploratory donor panel. Bamboo fibers were resistant to salivary, gastric, and intestinal enzymatic hydrolysis, as demonstrated by the absence of structural modifications in FTIR spectra, preserved morphology observed by scanning electron microscopy, and negligible release of reducing sugars (<0.1 g/L across all digestive phases), in contrast to extensively hydrolyzed wheat starch used as a positive control. During in vitro colonic fermentation, microbial responses were strongly dependent on substrate availability. In nutrient-limited minimal medium, bamboo fiber supplementation increased gas production, acidification, and SCFA formation compared with control conditions. Concomitantly, SCFA concentrations increased under minimal-medium conditions, although the magnitude of the response varied among donors, with the strongest changes observed for donor 1 (acetate reaching 5.4 vs. 3.3 g/L and propionate 1.48 vs. 0.76 g/L). These effects were markedly attenuated in nutrient-rich medium, indicating competition with readily fermentable substrates. Beta-diversity analyses and metagenomic profiling revealed that microbial community composition clustered primarily according to donor identity rather than experimental conditions. SEM further showed surface erosion and microbial attachment on fermented fibers, supporting partial structural alteration. Overall, bamboo fibers are resistant to upper gastrointestinal digestion but display context-dependent fermentative activity by human gut microbiota under carbohydrate-limited conditions, highlighting the importance of inter-individual variability, dietary context and substrate availability in determining their fermentative potential.

## 1. Introduction

Bamboo-derived products are attracting increasing interest as potential sources of sustainable dietary fiber for incorporation into food formulations [1]. Dietary fiber is recognized as a key modulator of gut microbiota, notably through the production of short-chain fatty acids (SCFAs), which contribute to host health [2,3,4]. However, the widespread adoption of Western dietary patterns, typically characterized by low fiber intake and high consumption of processed foods, has been associated with reduced microbial diversity and altered metabolic activity [5,6]. This context highlights the need to identify novel, sustainable, and functional sources of dietary fiber capable of restoring or modulating gut microbial activity and diversifying dietary fiber sources.

Among these, bamboo fibers are emerging as a promising but still underexplored candidate. Their increasing incorporation into food products worldwide, including in Europe, raises important questions regarding their digestibility and fermentability. Understanding the fermentation of bamboo fibers is particularly relevant for their potential incorporation into fiber-enriched foods, where microbial accessibility can influence both nutritional and functional properties. While several studies conducted using fecal microbiota from Asian donors have investigated bamboo fiber fermentation [7,8], the impact of dietary and environmental conditions on bamboo fiber utilization remains poorly understood. In particular, the contribution of substrate availability and microbial ecological factors to bamboo fiber fermentation remains insufficiently characterized.

Differences in gut microbiota composition have been reported across populations with distinct dietary patterns, including differences in diversity, community structure, and fermentation-related metabolic capacities [9,10]. However, these variations reflect complex interactions between habitual diet, lifestyle, host factors, and microbial ecology rather than geographical origin alone. As bamboo-derived ingredients are increasingly appearing in European markets, evaluating their digestibility and fermentation under controlled experimental conditions is relevant to better understand their potential impact on gut microbial metabolism.

From a structural perspective, bamboo fibers exhibit a highly recalcitrant lignocellulosic organization, composed mainly of cellulose (~73.8%) embedded in a matrix of hemicellulose (~12.5%) and lignin (~10.5%) [11]. Cellulose consists of linear β-1,4-linked glucose chains forming highly ordered crystalline structures [12], while hemicellulose is more amorphous and heterogeneous and remains closely associated with cellulose through hydrogen and covalent bonds [13]. Lignin, a complex aromatic polymer composed of phenylpropanoid units, further reinforces this matrix and is generally considered a structural component limiting enzymatic accessibility [14]. Compared with common insoluble dietary fibers such as wheat bran, which contains accessible arabinoxylan and cellulose fractions [15], bamboo fibers exhibit a more compact lignocellulosic organization with a higher proportion of crystalline cellulose and lignin, potentially limiting microbial accessibility.

While certain gut bacteria (e.g., *Ruminococcus*, *Bacteroides*, *Roseburia*) are capable of degrading polysaccharides such as cellulose and hemicellulose [16,17,18,19,20], a recent study indicated that specific lignin fractions, particularly soluble or less-condensed lignin structures, may undergo partial microbial transformation and modulate gut microbial activity and SCFA production [21]. Therefore, the structural complexity of bamboo fibers suggests that their accessibility to colonic microbiota may be limited and highly dependent on environmental conditions, including substrate availability and microbial composition.

Although bamboo fibers are generally considered resistant to digestion in the upper gastrointestinal tract [22,23], exposure to digestive conditions (e.g., pH variations, enzymatic activity, bile salts) may still alter their structure and influence their accessibility to microbial enzymes. Therefore, assessing both gastrointestinal digestion and colonic fermentation is essential to understand their fate after ingestion. However, the combined evaluation of bamboo fiber resistance during gastrointestinal digestion and subsequent microbial fermentation remains poorly documented.

In addition, the nutritional environment of the colon may strongly influence fiber fermentation. Readily available carbohydrates from the diet can compete with complex polysaccharides for microbial utilization, potentially masking the fermentative potential of resistant fibers [24]. Comparing nutrient-rich conditions with carbohydrate-limited conditions therefore provides insight into the contribution of bamboo fibers as a fermentable carbon source, while distinguishing their fermentation potential from microbial activity supported by readily fermentable substrates. The standard medium was used to represent a nutrient-rich environment where microbial activity is supported by readily available substrates, whereas the minimal medium was designed to evaluate the ability of bamboo fibers to sustain microbial fermentation when alternative carbon sources are limited. This approach allows discrimination between general microbial activity and fermentation responses specifically associated with bamboo fiber availability.

Therefore, this study aimed to evaluate the resistance of bamboo fibers to in vitro gastrointestinal digestion and to assess their fermentability using fecal microbiota from three healthy human donors. Microbial activity, metabolite production, and compositional changes were investigated under nutrient-rich and carbohydrate-limited fermentation conditions to determine how substrate availability influences bamboo fiber utilization.

## 2. Materials and Methods

### 2.1. Materials

Bamboo fibers were obtained from SaporePuro (Torino, Italy), and wheat starch was purchased from Cooper (Melun, France). α-Amylase, pepsin, lipase, potassium sodium tartrate, pancreatin, and DNS reagent were all supplied by Sigma-Aldrich (St. Louis, MO, USA). Bile extract was purchased from Thermo Scientific (Waltham, MA, USA), and sodium hydroxide was obtained from Prolabo (Paris, France). DNA extraction was performed using the QIAamp Fast DNA Stool Mini Kit^®^ (Qiagen, Hilden, Germany).

### 2.2. Methods

#### 2.2.1. In Vitro Gastrointestinal Simulated Enzymatic Digestion

##### Three-Phase Digestion

To assess the digestibility of bamboo fibers under simulated human gastrointestinal conditions, fibers were subjected to a modified INFOGEST in vitro digestion protocol based on the standardized consensus method described by Brodkorb et al. [25]. Deviations from the original protocol, including extended salivary, gastric and intestinal incubation times, were selected to better characterize the resistance of a highly insoluble fiber matrix to prolonged enzymatic exposure. The digestive fluids employed were Simulated Salivary Fluid (SSF), Simulated Gastric Fluid (SGF), and Simulated Intestinal Fluid (SIF). Starch was included as a positive control due to its well-characterized enzymatic digestibility [26,27], allowing verification that the digestive conditions and enzymatic activities were sufficient to induce carbohydrate hydrolysis. The transformation of bamboo fibers during digestion was subsequently analyzed to evaluate their structural stability and susceptibility to enzymatic hydrolysis.

- Oral digestion: Due to the high water-holding capacity and bulky structure of bamboo fibers, a maximum of 0.5 g of bamboo fibers could be used per digestion assay to ensure proper dispersion, efficient enzyme-substrate contact, and reproducible mixing throughout the digestion process. Higher amounts resulted in poor homogenization and limited accessibility of the digestive enzymes. The fibers were suspended in 4.5 mL of distilled water. Therefore, 5 g of wheat starch, a readily digestible carbohydrate matrix, was processed in parallel as a positive control. For each sample, 3.75 mL of SSF and 5 µL of CaCl_2_, 2H_2_O (44.1 g/L) were added, followed by 1.2 mL of alpha-amylase (625 U/mL). The final α-amylase activity in the oral digestion mixture was approximately 75 U/mL. Samples were incubated for 5 min at 37 °C, 50 rpm and then placed in a 100 °C water bath for enzyme inactivation.
- Gastric digestion: For gastric digestion, 5 g of salivary digestate was mixed with 3.75 mL of SGF. The pH was adjusted to 3, then 5 µL of CaCl_2_, 2H_2_O (44.1 g/L) was added as well as 324 µL of pepsin (61,815.6 U/mL) and 370 µL of lipase (1617 U/mL). The final enzymatic activities in the gastric phase were 2000 U/mL for pepsin and 60 U/mL for lipase. The pH was readjusted to 3 and the final volume was brought to 10 mL with distilled water. Samples were incubated for 4 h at 37 °C, 50 rpm and then placed in a 100 °C water bath for enzyme inactivation.
- Intestinal digestion: For intestinal digestion, 5 g of gastric digestate was mixed with 3.75 mL of SIF. The pH was adjusted to 7. Then, 500 µL of bile solution (200 mM), 20 µL of CaCl_2_, 2H_2_O (44.1 g/L), and 500 µL of pancreatin (equivalent to 2000 U/mL trypsin activity) were added; then the pH was adjusted again to 7, and the final volume was made up to 10 mL with distilled water. Pancreatin was added to reach a final trypsin activity of 100 U/mL in the intestinal digestion mixture. The samples were incubated for 6 h at 37 °C, 50 rpm and then placed in a 100 °C water bath for enzyme inactivation.
- Undigested controls: At each digestion phase, additional samples were prepared as described previously but were not incubated. They were directly placed in the 100 °C water bath for 10 min to inactivate the enzymes. These samples served as undigested controls for subsequent analysis.

All digestion assays were performed in technical triplicate to ensure reproducibility.

##### Fourier Transform Infrared Spectroscopy (FTIR)

To compare the structure of samples subjected to enzymatic digestion with that of undigested controls, an infrared spectrophotometer (IRAffinity-1S IRTF, Shimadzu, Kyoto, Japan) was used at each digestion stage. This technique enables the identification and monitoring of potential changes in characteristic functional groups, confirming the formation or disappearance of specific chemical bonds related to enzymatic digestion [28,29]. The spectrophotometer was equipped with an attenuated total reflectance accessory (ART-IRTF, Miracle 10). The absorbance of individual components was recorded from 400 to 4000 cm^−1^, at room temperature and over 50 scans at 4 cm^−1^ resolution using Happ-Genzel transmittance.

##### Quantification of Reducing Sugars (DNS Assay)

Polymer digestion results in the release of monomers or reducing sugars, the quantification of which allows for the assessment of the extent and efficiency of the digestive process. The quantification of reducing sugars was performed by applying the 3.5 dinitro salicylic acid (DNS) method [30]. The reagent was prepared as follows: 30 g of potassium sodium tartrate and 1.6 g of sodium hydroxide were prepared in 100 mL of distilled water and then the solution was poured into a bottle containing 1 g of DNS. The assay was carried out with 100 µL of sample to which 100 µL of DNS reagent was added, the mixture was vortexed and then placed in a dry bath at 100 °C for 5 min and then placed in ice. 1 mL of water was added to the tubes and then the absorbance reading was carried out with a spectrophotometer at 540 nm. The calibration curve was carried out with glucose samples at different concentrations; the linearity of the quantification curve achieved an R^2^ value of 0.9992. The results were expressed as glucose equivalents (g/L of digestion medium). Considering the different initial substrate loads used for bamboo fibers and wheat starch, reducing sugar release was additionally normalized to the initial amount of substrate (mg glucose equivalents per g of substrate) to allow a more appropriate comparison between samples.

##### Scanning Electron Microscope (SEM) Observations During Digestion

Fibers were observed under a scanning electron microscope at each stage of digestion. Samples were fixed overnight at 4 °C with 2.5% glutaraldehyde and 2% paraformaldehyde in 0.2 M sodium cacodylate buffer, pH 7.4. They were washed in sodium cacodylate buffer and post-fixed for 1 h with 1% osmium tetroxide in the same buffer.

After rinsing for 20 min in distilled water, dehydration by graded ethanol was performed from 70° to 100° (10 min each) to finish in hexamethyldisilazane (HMDS) for 10 min. Samples were deposited on stubs using adhesive carbon tabs and coated with platinum Quorum Q150 TES (Quorum Technologies, Laughton, UK). Observations were carried out using a Scanning Electron Microscope Jeol 6060 LV (JEOL Ltd., Tokyo, Japan) at 5 kV with a secondary electron detector. Representative SEM images are shown for clarity; similar structural features were consistently observed across all replicates and samples.

#### 2.2.2. In Vitro Colonic Fermentation

##### Fecal Inoculum

To investigate the microbial degradability of bamboo fibers in the human colon, in vitro fermentation was performed using human fecal microbiota to simulate colonic conditions. Fecal samples were collected from three healthy donors: donor 1, a 27-year-old woman consuming a typical Western diet; donor 2, a 63-year-old man consuming a Western diet rich in fiber; and donor 3, a 29-year-old woman also consuming a Western diet rich in fiber. None of the donors had received antibiotic or probiotic treatment for at least two months prior to the study. All participants provided written, informed consent, and the use of human fecal material was approved by the French Ethical Research Committee (Comité de protection des personnes (CPP) Sud-Est VI, Clermont-Ferrand, France) under registration number 22.03666.000109-MS01. Stool samples were collected and kept anaerobically for a maximum of 2 h at 37 °C before inoculation. Then, they were processed under strict anaerobic conditions (PureEvo T4, Jacomex, Dagneux, France). The stools were tenfold diluted in a phosphate buffer (30 mM, pH = 6.5) + cysteine (1.9 mM), then ground to homogenize the suspension with an immersion blender and then the suspension was filtered through a 500 µm sieve.

##### Fermentation

In vitro fermentations were carried out using four different media to assess the effect of bamboo fibers on microbial metabolism. Anaerobic flasks (total volume of 60 mL) were prepared under nitrogen flushing. The media included a standard medium, containing various carbohydrate, protein, lipid, mineral, and vitamin sources, as previously described [31], a standard medium supplemented with 13 g/L of bamboo fibers (standard medium + bamboo), a minimal medium without any of the carbohydrates or fibers originally included (minimal medium), and a minimal medium supplemented with 13 g/L of bamboo fibers (minimal medium + bamboo). The concentration of bamboo fibers was chosen to match the total amount of sugars and fibers present in the standard medium. This last condition was tested to evaluate the behavior of gut microbiota when bamboo fibers served as the sole carbon source. This design allowed the assessment of bamboo fiber fermentability under both nutrient-rich and nutrient-limited conditions and the monitoring of microbial activity and metabolite production in response to fiber supplementation.

Each flask was filled with 25 mL of medium. In each flask, 2.8 mL of the previously described stool solution was added, the suspension was homogenized and then 1 mL of medium was taken to measure the initial pH. The flasks were incubated for 48 h at 37 °C, 90 rpm. The 48 h endpoint was selected based on preliminary kinetic tests showing stabilization of gas production and SCFA accumulation after 24–36 h, indicating a late fermentation phase representative of fiber degradation under in vitro colonic conditions. The pH was monitored twice daily and, when necessary, adjusted to pH 7.0. This adjustment was implemented to avoid excessive acidification during batch fermentation, which lacks the continuous pH regulation present in dynamic in vitro colon models such as Human Intestinal Microbial Ecosystem (SHIME^®^) or human ARtificial COLon model (ARCOL) [32,33]. In these systems, colonic pH is maintained within physiological ranges by continuous automatic regulation. In our simplified batch model, intermittent adjustment to pH 7.0 was used as a practical compromise to limit excessive pH drift between measurements while maintaining microbial activity throughout the incubation period. The reported pH decreases correspond to the difference between pH 7.0 and the pH measured immediately before each scheduled readjustment. Once a day, the gas composition of the gaseous part of the flasks was analyzed. At the end of the experiment, the metabolite composition was analyzed by HPLC. Each condition was carried out in triplicate for each donor. Anaerobic conditions were maintained throughout the experiment, and additions or withdrawals from both the liquid and gaseous phases were performed using syringes and needles.

##### Gas Analysis

The gases present in the gaseous fraction of the flasks were analyzed to assess the metabolism of the microorganisms in the presence or absence of fibers. Relative abundances of CO_2_, CH_4_, and H_2_ present in the flask atmospheric phase were determined using a 490 micro-gas chromatography (Agilent Technologies, Santa Clara, CA, USA) equipped with a Molecular Sieve 5Å column and a PoraPlot U column coupled with thermal conductivity detector TCD. Argon was used as the carrier gas. Calibration curves were made from four gases: (i) ambient air (78% N_2_, 21% O_2_ and 0.04% CO_2_), (ii) mixture A (5% CO_2_, 5% H_2_ and 90% N_2_), (iii) mixture B (20% CO_2_ and 80% H_2_), and (iv) mixture C (19.9% CO_2_, 19.9% CH_4_, 20% H_2_ and 40% N_2_). Results were expressed as relative percentages.

##### SCFA Analysis

Short-chain fatty acids (SCFAs) were determined using a liquid chromatograph (1260 HPLC, Agilent, Santa Clara, CA, USA) to assess the metabolic activity of microorganisms by providing direct indicators of their metabolic production. The HPLC apparatus was equipped with two columns (Rezex ROA 300 mm × 7.8 mm, Phenomenex, Torrance, CA, USA) connected in series in an oven (50 °C) and coupled with a refractive index detector. The mobile phase was 2 mM sulfuric acid in ultra-pure water pumped at 0.7 mL/minute and 70 bars. For the analysis, 2 mL of sample was mixed with 250 µL of Ba(OH)_2_·8H_2_O (0.3 M) and 250 µL of ZnSO_4_·7H_2_O (5% *w*/*v*) before a 5 min centrifugation at 10,000 *g*. Samples were filtered through 0.2 µm nylon filters before being injected into the HPLC system.

##### DNA Extraction

To characterize the microbial species developed under the different tested conditions, DNA extractions were performed on either the liquid fractions, the liquid + fiber fractions, or the washed fibers alone. For this purpose, 2 mL of the medium containing fibers was collected, filtered through a 500 µm sieve, and washed twice with sterile saline solution. Finally, 200 mg of washed fibers were recovered, and DNA extraction was performed on these fibrous samples to determine if any species remained attached to the fibers and, if so, to identify them. Genomic DNA was extracted using the QIAamp Fast DNA Stool Mini Kit^®^. 2 mL of liquid samples or 200 mg of fibers were vortexed and centrifuged (1 min, 8000 *g*) before being ground in the BeadBeater (5 min, 20 beats/s) with 300 mg of sterile beads (diameter ranging from 0.1 to 0.6 mm) and 1 mL InhibitEx Buffer^®^ from Qiagen kit. Then, DNA was extracted following the manufacturer’s instructions. DNA quantity was measured with the NanoDrop™ 2000 Spectrophotometer (Thermo Scientific™). DNA samples were stored at −20 °C prior to shotgun metagenomics analysis.

##### Shotgun Metagenomics and Data Analysis

Libraries were prepared using the sparQ DNA Fragment and Library Prep kit (Avantor, Radnor, PA, USA; Cat. No. 76183-244) from 8 ng of genomic DNA per sample, following the manufacturer’s instructions, with a 10 min fragmentation step. Fragments were ligated with Quantabio UDI adapters (diluted 1:25) for sample indexing. After 18 cycles of final amplification, libraries were purified using AMPure XP beads (Beckman Coulter, Brea, CA, USA) and resuspended in 30 µL. Library quality was assessed by capillary electrophoresis on a QIAxcel Advanced system (Qiagen) using a high-resolution cartridge (Cat. No. 1050450). Libraries were pooled in equimolar quantities based on capillary electrophoresis signal intensity at approximately 500 bp (corresponding to the fragmentation size plus adapter length). The pooled library was purified using the GeneJET PCR Purification Kit (Thermo Fisher Scientific, Waltham, MA, USA, Cat. No. K0702), and fragments of 500 bp ± 8% were size-selected by electrophoresis on a PippinHT system (Sage Science, Beverly, MA, USA) using a 1.5% agarose cassette. Fragment size and concentration of the purified pool were confirmed by capillary electrophoresis (QIAxcel) and fluorometric quantification (Qubit 4.0, Thermo Fisher Scientific), respectively. Library quality was first verified on a MiSeq instrument (Illumina, San Diego, CA, USA) using 2 × 251 bp paired-end reads (MiSeq Reagent Kit v2, 302 cycles), with loading concentrations targeting a theoretical cluster density of 800–1000 K/mm^2^. Following this verification, quantities were adjusted and the pooled library was sequenced on a NovaSeq 6000 instrument (Illumina) using 2 × 151 bp paired-end reads.

Demultiplexed reads were quality-checked using FastQC (v0.12.1), with results compiled using MultiQC, and trimmed and filtered using fastp. Across samples, 113,446–5,285,979 reads were obtained before filtering and 100,287–5,215,257 reads after quality filtering and contaminant removal. Potential host and PhiX reads were removed using Bowtie2 with default parameters against the human (GRCh38.p14), mouse (GRCm39), rat (GRCr8), and PhiX (GCF_000819615.1) reference genomes. Taxonomic affiliation was performed using MetaPhlAn 4.0 with default parameters against the vJan25_CHOCOPhlAnSGB_202503 database, and resulting taxonomy was converted to GTDB format. MetaPhlAn 4.0 provided relative abundances normalized to 100% per sample. For downstream QIIME 2 analyses, these values were multiplied by 1000 and converted to integer pseudo-counts. Samples were rarefied to 9000 reads using the QIIME 2 core-metrics workflow; rarefaction curves confirmed a plateau in observed species richness at this depth.

Alpha diversity (Shannon, Simpson) and beta diversity (Jaccard and Bray–Curtis distances) were computed using QIIME2. Jaccard distance was based on the presence or absence of detected microbial taxa, while Bray–Curtis dissimilarity accounted for differences in relative abundance. Distance matrices were exported and further analyzed in R (version 4.4.2) using the vegan package. Principal coordinate analyses (PCoA) were performed using the cmdscale function. The effects of donor identity, fermentation medium, and bamboo fiber supplementation on microbial community structure were assessed using PERMANOVA (999 permutations) with the adonis2 function. Homogeneity of multivariate dispersion was assessed using betadisper followed by ANOVA.

##### SEM Observations After Fermentation

Fibers were observed under the scanning electron microscope before and after fermentation by the intestinal microbiota to observe the effects of fermentation on fiber morphology as described in Section 2.2.1.

#### 2.2.3. Statistical Analysis

Data were expressed as means ± SD in triplicate. GraphPad Prism Version 10.5.0 for MacOS, GraphPad Software, Boston, MA, USA, was used for statistical analysis. For the in vitro digestion experiments, differences between substrates and digestion phases were analyzed using two-way ANOVA followed by Sidak’s multiple-comparison test. Gas production and pH measurements were analyzed using two-way ANOVA followed by Tukey’s multiple-comparison test. Alpha diversity indices were analyzed using one-way ANOVA followed by Tukey’s multiple-comparison test. Each experimental condition was performed in technical triplicate for each donor. Statistical analyses were conducted independently for each donor and were therefore intended to evaluate within-donor responses rather than population-level effects. Statistical significance was set at *p* < 0.05.

## 3. Results

### 3.1. Bamboo Fibers Resist Enzymatic Digestion

#### 3.1.1. FTIR Analysis

**Figure 1:**
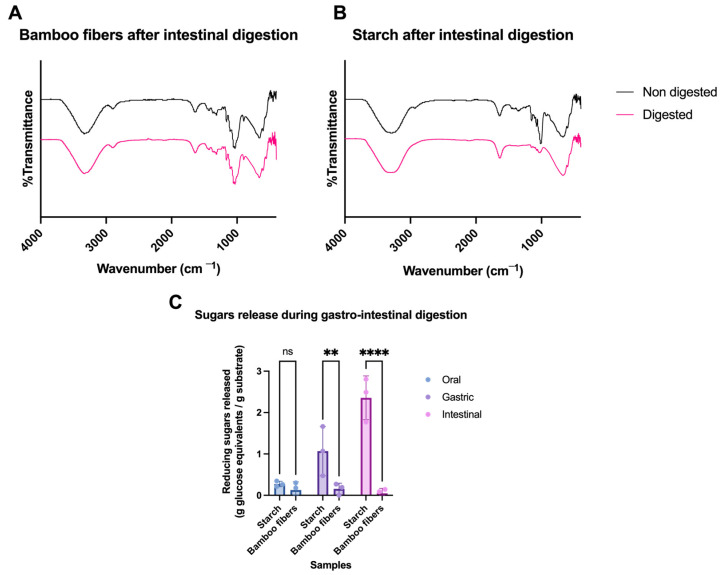
Structural and biochemical assessment of bamboo fiber digestion. (**A**) FTIR spectra of bamboo fibers before and after simulated gastrointestinal digestion showing no structural modification. (**B**) FTIR spectra of starch highlighting the disappearance of the band at ~1150 cm^−1^ after digestion. (**C**) Quantification of reducing sugars released during oral, gastric, and intestinal phases for bamboo fibers and starch. All values are mean ± standard deviation. *n* = 3. ns means non-significant, ** means *p* < 0.01 and **** means *p* < 0.0001.

FTIR analysis of bamboo fibers showed no noticeable difference between undigested and digested samples. The spectra fully overlap, indicating that the chemical structure of bamboo fibers remains unchanged after exposure to salivary, gastric, and intestinal enzymes (Figure 1A). This suggests that bamboo fibers are not hydrolyzed by gastrointestinal enzymes and retain their structural integrity throughout the digestion process.

Under the same conditions, digested wheat starch exhibited a clear spectral modification (Figure 1B), with the disappearance of a characteristic band at approximately 1150 cm^−1^. This change was consistent with enzymatic hydrolysis of glycosidic linkages in starch and confirms that the digestion protocol is functional and discriminative. The rest of the FTIR spectrum remained largely similar, corresponding to the breakdown of the crystalline starch structure while maintaining some underlying chemical features of the polysaccharide backbone.

#### 3.1.2. Quantification of Reducing Sugars

Enzymatic digestion of starch is known to release large amounts of reducing sugars as α-amylase, pepsin (indirectly), and pancreatic enzymes progressively cleave its glycosidic bonds [34]. If bamboo fibers were susceptible to gastrointestinal digestion, reducing sugars would also be expected to be released during the oral, gastric, and intestinal phases. Therefore, reducing sugars were quantified throughout digestion to assess carbohydrate hydrolysis. Because different initial substrate masses were required for starch (5 g) and bamboo fibers (0.5 g), reducing sugar release was normalized to the initial substrate mass to enable comparison between substrates. As expected, starch underwent progressive hydrolysis throughout digestion, with reducing sugar release increasing from 0.80 ± 0.07 g glucose equivalents/g substrate after the oral phase to 1.07 ± 0.60 g/g after the gastric phase and 2.36 ± 0.53 g/g after the intestinal phase (Figure 1C). In contrast, bamboo fibers released only negligible amounts of reducing sugars throughout digestion (0.13 ± 0.20, 0.16 ± 0.14, and 0.05 ± 0.12 g glucose equivalents/g substrate after the oral, gastric, and intestinal phases, respectively), with no significant increase across digestive phases. Reducing sugar release was significantly higher for starch than for bamboo fibers during the gastric phase (*p* = 0.0071) and the intestinal phase (*p* < 0.0001). These findings indicated that bamboo fibers were highly resistant to enzymatic hydrolysis under the simulated gastrointestinal conditions.

#### 3.1.3. SEM Observations

**Figure 2:**
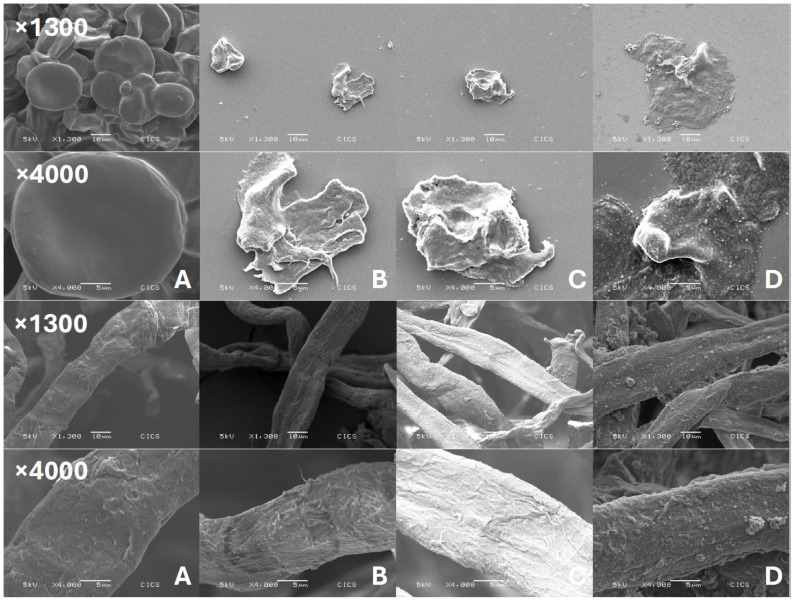
SEM observations of starch grains (**top**) and bamboo fibers (**bottom**) before digestion (**A**), after salivary digestion (**B**), after gastric digestion (**C**), and after intestinal digestion (**D**). Images are presented at two magnifications: ×1300 (first and third rows) and ×4000 (second and fourth rows).

The structural integrity of bamboo fibers was also assessed by microscopic observations to identify any potential structural modifications induced by enzymatic digestion. Their elongated, fibrous structure remained intact throughout the different digestion stages, without signs of erosion, surface collapse, or fragmentation (Figure 2). These images corroborated both FTIR and reducing sugar concentration results, demonstrating that bamboo fibers maintained their physical structure and exhibited resistance to enzymatic hydrolysis during the 10 h digestion protocol.

In contrast, starch granules, which appeared intact prior to digestion, exhibited substantial morphological alterations following enzymatic treatment. Initially smooth, spherical grains (A) lost their structural integrity after salivary digestion (B), showing surface erosion. This degradation intensified during gastric (C) and intestinal phases (D), where granules appeared fragmented, collapsed, and amorphous (Figure 2). These observations confirmed the complete susceptibility of wheat starch to digestive enzymes.

### 3.2. Bamboo Fibers Are Utilized as Fermentable Substrate by the Human Gut Microbiota

#### 3.2.1. Fermentation Activity: Gas Production, pH Changes and SCFA Formation

**Figure 3:**
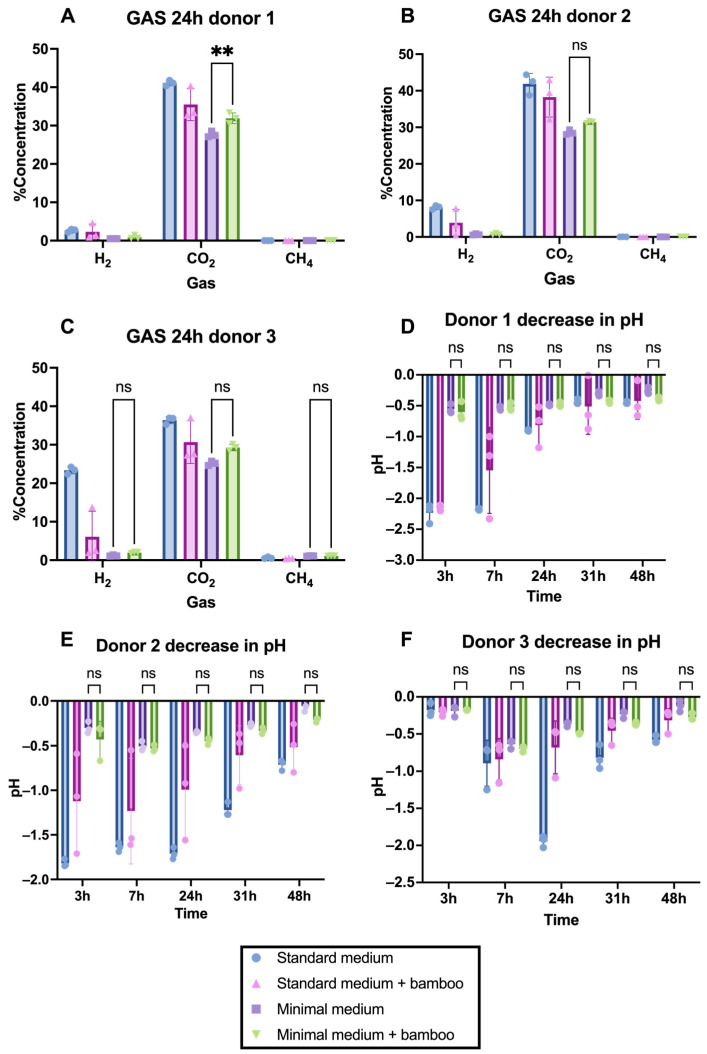
Fermentation activity of gut microbiota assessed by gas production and pH evolution in Standard and Minimal Media for three donors. (**A**–**C**) Gas production after 24 h of fermentation for donor 1 (**A**), donor 2 (**B**), and donor 3 (**C**). (**D**–**F**) pH decrease over time during fermentation for donor 1 (**D**), donor 2 (**E**), and donor 3 (**F**). Results are expressed as mean ± standard deviation (*n* = 3). Statistical significance is indicated as ns (not significant), ** *p* < 0.01.

**Figure 4:**
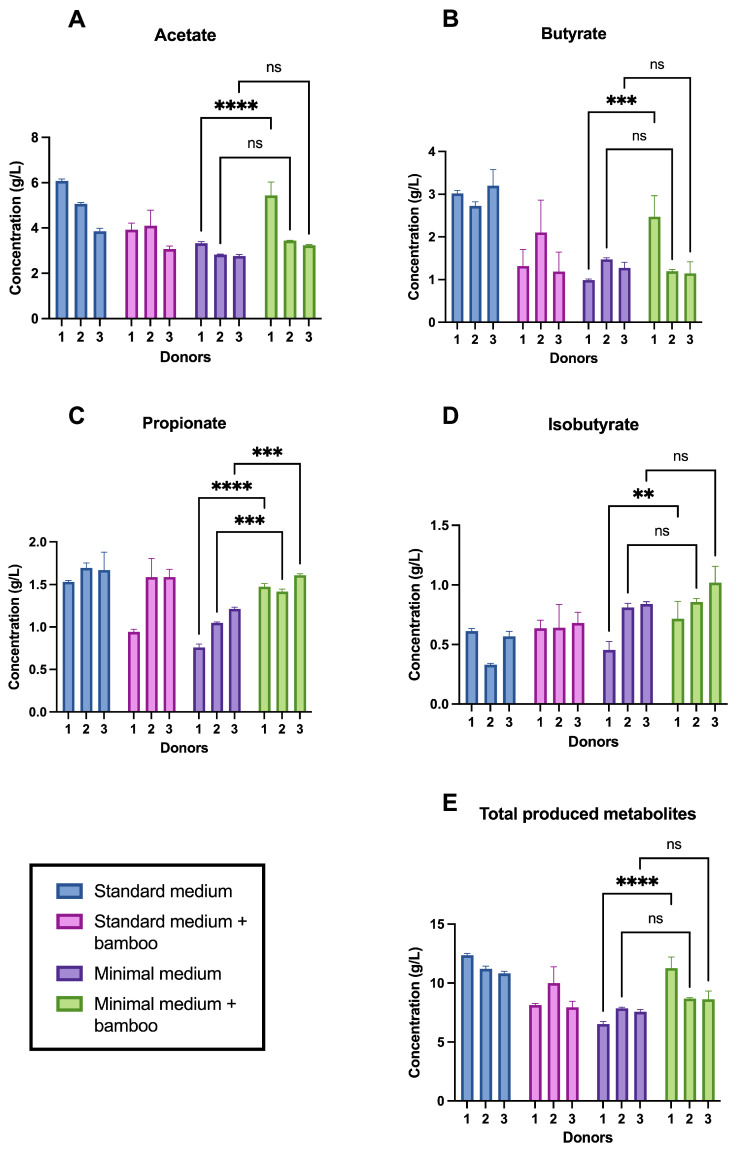
Short-chain fatty acids (SCFAs) and related metabolite production (g/L) after 48 h of fermentation for the three donors. (**A**) acetate, (**B**) butyrate, (**C**) propionate, (**D**) isobutyrate, and (**E**) total metabolite production. Results are expressed as mean ± standard deviation (*n* = 3). Statistical significance is indicated as ns (not significant), ** *p* < 0.01, *** *p* < 0.001, and **** *p* < 0.0001.

Microbial fermentation during incubation was assessed over 48 h through gas production (H_2_, CO_2_, CH_4_) and pH evolution (Figure 3A–F), together with short-chain fatty acid (SCFA) production (Figure 4). A nutrient-rich complete medium was used as a control to assess baseline microbial activity in the in vitro model prior to bamboo fiber fermentation experiments.

##### Gas Production

The fermentation of carbohydrates by the gut microbiota to produce SCFAs is commonly accompanied by gas formation. Dihydrogen (H_2_) production plays a key role in regenerating NAD^+^ from NADH during fermentation, while carbon dioxide (CO_2_) is generated during decarboxylation reactions [35]. The generated H_2_ may be utilized by methanogenic archaea as an electron donor for methane (CH4) production [36]. After 24 h, CO_2_ concentrations ranged from 30 to 40% across all conditions (Figure 3A–C). In standard medium (SM), containing readily fermentable carbohydrates, CO_2_ levels were higher than in minimal medium (MM), suggesting higher overall microbial activity [37]. In MM, bamboo supplementation increased CO_2_ and H_2_ production compared with MM alone, although the magnitude of the response varied among donors. Bamboo fibers increased CO_2_ levels in all three donors, reaching 31.9% versus 27.7% (*p* = 0.008) for donor 1, 31.4% versus 28.6% for donor 2, and 29.3% versus 25.2% (*p* = 0.007) for donor 3, although the effect was statistically significant only for donors 1 and 3. A similar pattern was observed for H_2_, with higher levels in the presence of bamboo (0.89% versus 0.52% for donor 1, 0.72% versus 0.68% for donor 2, and 1.96% versus 1.19% for donor 3), but a significant difference was detected only for donor 3. Methane production in donor 3 was higher in MM than SM, suggesting donor-specific substrate utilization rather than a direct effect of bamboo fibers.

##### pH Evolution

Bacteria in the gut microbiota utilize fiber through fermentation, producing SCFAs that contribute to the acidification of the intestinal environment [37]. During fermentation, the pH thus decreased in all conditions for the three donors (Figure 3D–F).

Acidification mirrored gas production trends. In SM, pH decreased rapidly due to abundant fermentable carbohydrates, and bamboo fiber addition had minimal effect. In MM, pH decreased more gradually. For example, at 31 h, a greater decrease in pH was observed in the presence of bamboo fibers compared with MM alone, corresponding to ~43% and ~57% higher acidification for donors 1 and 3, respectively (−0.43 vs. −0.30 and −0.36 vs. −0.23). The presence of bamboo fibers led to slightly greater reductions than MM alone, though differences were not statistically significant. This gradual acidification was consistent with microbial utilization of bamboo fibers under substrate-limited conditions.

##### SCFA Production

Bamboo fiber supplementation in MM promoted SCFA formation, with donor-specific responses (Figure 4). Acetate concentrations were higher in the presence of bamboo for all donors, with statistically significant differences observed for donor 1 (5.4 g/L vs. 3.3 g/L, *p* < 0.0001). Propionate levels also increased with bamboo supplementation, reaching statistical significance for all donors, with a stronger effect observed for donor 1 (1.48 g/L vs. 0.76 g/L, *p* < 0.0001) compared to donors 2 and 3. Butyrate production was significantly increased in donor 1, while only moderate, non-significant increases were observed in donors 2 and 3 (1.48 g/L vs. 1.20 g/L and 1.28 g/L vs. 1.14 g/L respectively). Isobutyrate showed a significant increase only in donor 1, with no significant changes in the other donors. Total SCFA production was significantly higher in donor 1 in the presence of bamboo, whereas donors 2 and 3 exhibited similar increasing trends that did not reach statistical significance, confirming that bamboo fibers can serve as a fermentable substrate under carbohydrate-limited conditions, with responses depending on donor microbiota composition.

In SM, SCFA concentrations were largely driven by the readily fermentable carbohydrates present in the medium, and bamboo supplementation had minimal impact, consistent with the patterns observed for gas production and pH.

Together, the results indicated that bamboo fibers resist upper gastrointestinal digestion but can support microbial fermentation under nutrient-limited conditions, although fermentation intensity varied according to donor-specific microbiota composition. Bamboo fibers contributed to increased gas production, gradual acidification, and SCFA formation in MM, while their effect was limited in the presence of readily fermentable sugars. Inter-donor variability highlighted the influence of individual microbiota composition and substrate availability on fermentation outcomes.

#### 3.2.2. Fiber Degradation and Microbial Community Structure

##### Morphological Changes in Bamboo Fibers After Fermentation

**Figure 5:**
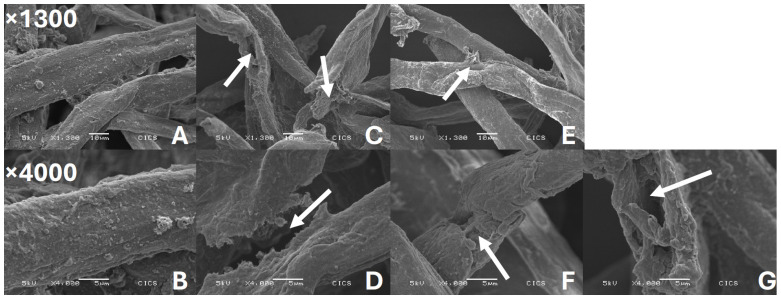
SEM observations of bamboo fibers before digestion (**A**,**B**) and after gut microbiota fermentation (**C**–**G**) at ×1300 (**top**) and at ×4000 (**bottom**). White arrows indicate damaged areas and structural alterations observed after fermentation.

SEM analysis revealed structural alterations of bamboo fibers following fermentation (Figure 5). Holes and tears appeared on the fiber surface, which were absent in unfermented controls. These morphological changes suggest that bacterial activity may induce partial modification of bamboo fiber surfaces. These morphological changes were consistent with the fermentation activity observed through SCFA formation and gas production. The observed alterations suggested partial structural modification of bamboo fibers during fermentation, although quantitative fiber degradation was not directly measured.

##### Microbial Alpha Diversity Across Experimental Conditions

**Figure 6:**
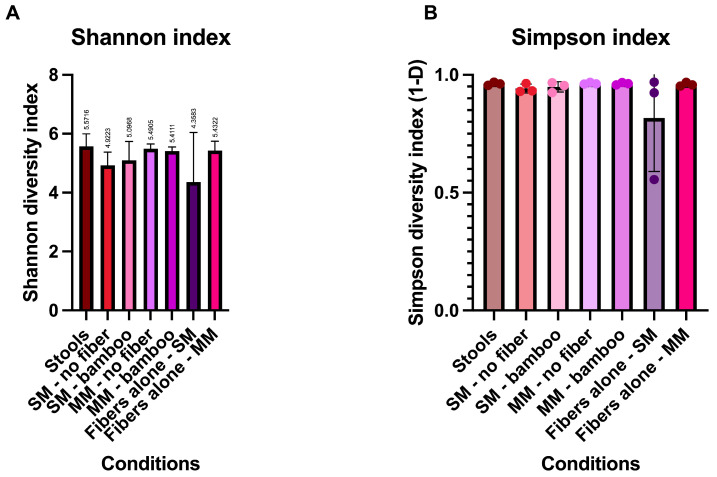
Alpha diversity of microbial communities. (**A**) Shannon diversity index and (**B**) Simpson diversity index (1-D) across experimental groups. SM = Standard Medium; MM = Minimal Medium. All values are expressed as mean ± standard deviation (*n* = 3).

The alpha diversity of microbial communities was assessed using the Shannon and Simpson indices. The Shannon index reflects both species richness and evenness, while the Simpson index (1-D) emphasizes dominance and abundance distribution. These complementary indices allow for the characterization of overall diversity and community structure within each sample. Most samples had Shannon indices between 4.3 and 5.6, reflecting species richness and a relatively balanced distribution. Simpson indices (1-D), around 0.96, indicated that no single taxon dominates the communities, thus confirming a balanced distribution of species (Figure 6). One-way ANOVA tests revealed no significant differences (*p* > 0.05) in alpha diversity (Shannon or Simpson indices) between the different experimental conditions. Overall, the addition of fibers and the absence of sugars were not associated with reduced microbial alpha diversity, as Shannon and Simpson indices remained high across all conditions.

##### Microbial Beta Diversity Across Experimental Conditions

**Figure 7:**
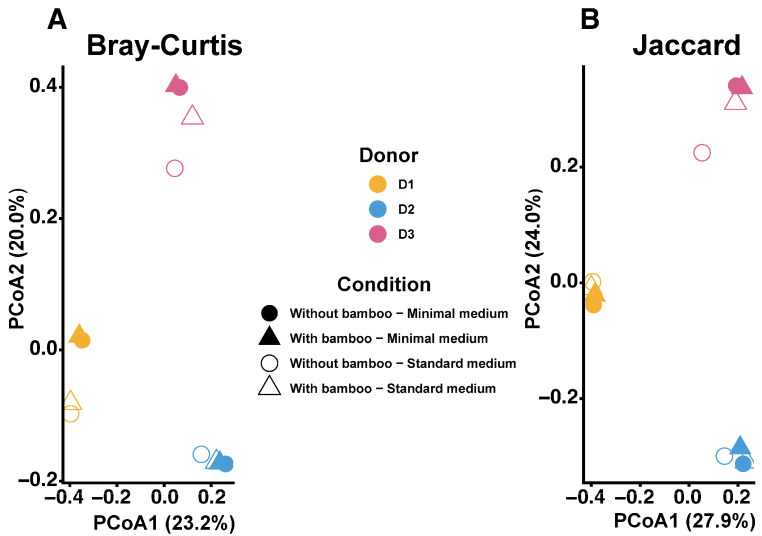
(**A**) Principal coordinate analysis (PCoA) based on Bray–Curtis dissimilarity and (**B**) PCoA based on Jaccard distance. Samples are colored according to donor identity and shaped according to experimental conditions (bamboo supplementation and culture medium).

Beta diversity was assessed using Bray–Curtis dissimilarity and Jaccard distance matrices to evaluate differences in microbial community composition between samples based on abundance and presence/absence patterns, respectively. Principal coordinate analyses (PCoA) revealed a clear separation of samples according to donor identity, with samples clustering primarily by donor rather than by experimental conditions (Figure 7). Samples from the same donor remained closely grouped regardless of bamboo supplementation or medium composition, although slight shifts between conditions were observed within individual donors. For Bray–Curtis distances, the first two principal coordinates explained 43.2% of the total variation (PCoA1: 23.2%; PCoA2: 20.0%), whereas Jaccard-based PCoA explained 51.8% of the variation (PCoA1: 27.9%; PCoA2: 24.0%).

PERMANOVA analyses confirmed that donor identity was the main factor driving microbial community variation. When donor identity was included in the model, it explained 81.2% of the variance in microbial community structure for both Bray–Curtis (R^2^ = 0.81159, *p* = 0.001) and Jaccard (R^2^ = 0.81233, *p* = 0.001) distances. In contrast, bamboo supplementation and medium composition did not significantly affect overall community structure when donor variability was considered (*p* > 0.05).

Homogeneity of multivariate dispersion was assessed using betadisper analysis. No significant differences in dispersion were observed between medium conditions or between bamboo and non-bamboo conditions for either Bray–Curtis or Jaccard distances (*p* > 0.05), confirming that PERMANOVA results were not driven by differences in within-group variability.

##### Shifts in Microbial Composition

**Figure 8:**
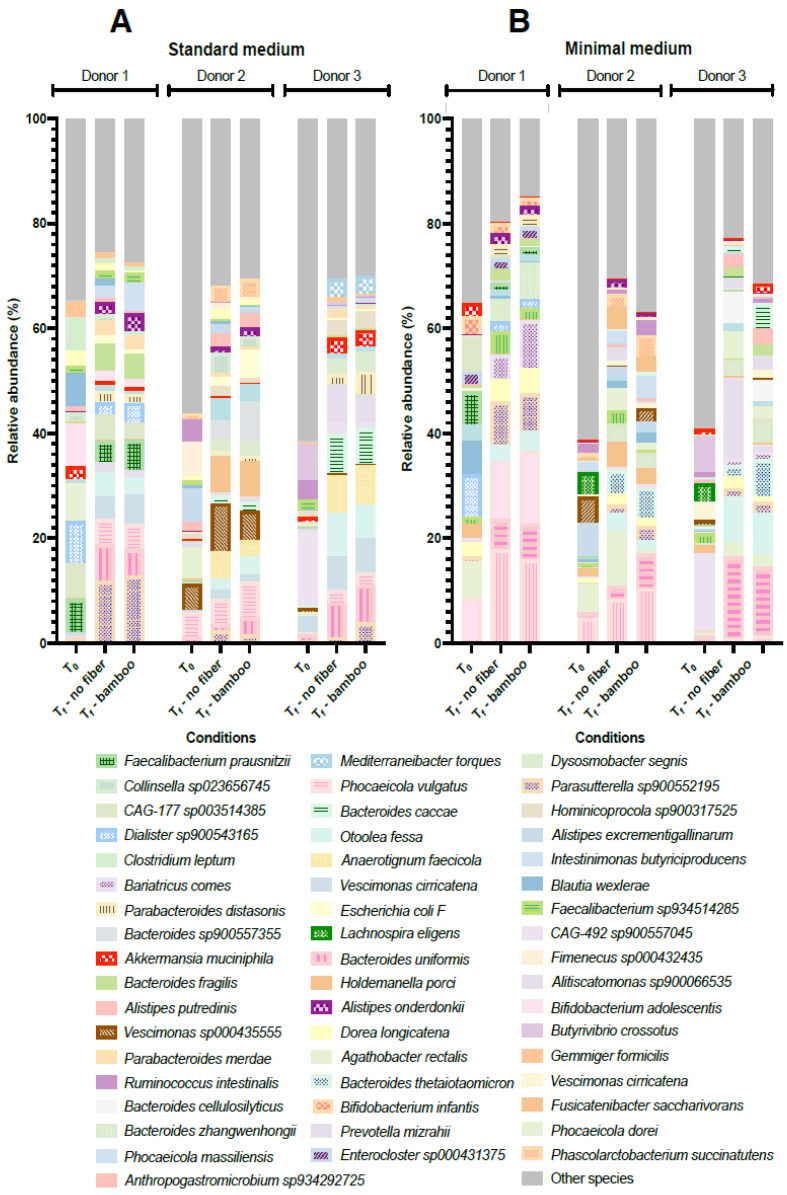
Relative abundance of the top 40 microbial species at initial (T_0_) and final (T_F_) time points under different fermentation conditions. (**A**) Standard Medium; (**B**) Minimal Medium, each with or without bamboo fibers.

Metagenomic analysis revealed dynamic changes in microbial community composition between t_0_ and t_f_, which varied according to donor and experimental condition (Figure 8). These shifts varied according to donor identity and experimental conditions, highlighting the influence of substrate availability on specific microbial responses rather than overall community structure.

- Standard medium (Figure 8A):

Bamboo supplementation was associated with modest shifts in the relative abundance of several taxa in two of the three donors. For example, *Akkermansia muciniphila* represented a higher proportion of the community after fermentation with bamboo (from 0.4% to 1.7% and from 0.1% to 0.2%), while *Bacteroides thetaiotaomicron* and *Bifidobacterium adolescentis* also showed increased relative abundances (from 2% to 7.1%, 4.4% to 5.6%, and 11.1% to 13.8%, respectively). Other species, including *Bacteroides uniformis*, *Phocaeicola vulgatus*, *Bacteroides caccae*, and *Bacteroides zhangwenhongii*, displayed similar compositional shifts. Overall, these compositional changes were limited, likely reflecting the preferential utilization of readily fermentable substrates present in SM.

- Minimal medium (Figure 8B):

Bamboo supplementation in MM was associated with more pronounced changes in the relative abundance profiles of several taxa across donors. The relative abundance of *Intestinimonas butyriciproducens* increased in all three donors (from 2.3% to 5.3%, 0.1% to 0.2%, and 0.4% to 0.9%). Additional increases in relative abundance were observed in *Parabacteroides distasonis* (2.0% to 4.4% and 0.7% to 1.0%), *Bacteroides uniformis* (6.5% to 7.0% and 1.3% to 3.3%), *Alistipes onderdonkii* (2.3% to 3.5% and 1.3% to 1.7%), *Phascolarctobacterium succinatutens* (2.9% to 3.3%), and *Faecalibacterium prausnitzii* (4.1% to 5.8%). These taxa showed higher relative abundances in bamboo-supplemented conditions, although their direct involvement in bamboo fiber degradation cannot be inferred from relative abundance data alone.

Overall, these results demonstrated that bamboo fibers were associated with donor- and condition-specific changes in microbial community composition, with stronger responses observed under carbohydrate-limited conditions (MM). The observed shifts were consistent with enhanced fermentation activity, as indicated by gas production, pH decrease, and SCFA formation.

##### Fiber-Attached Versus Planktonic Microbiota

**Figure 9:**
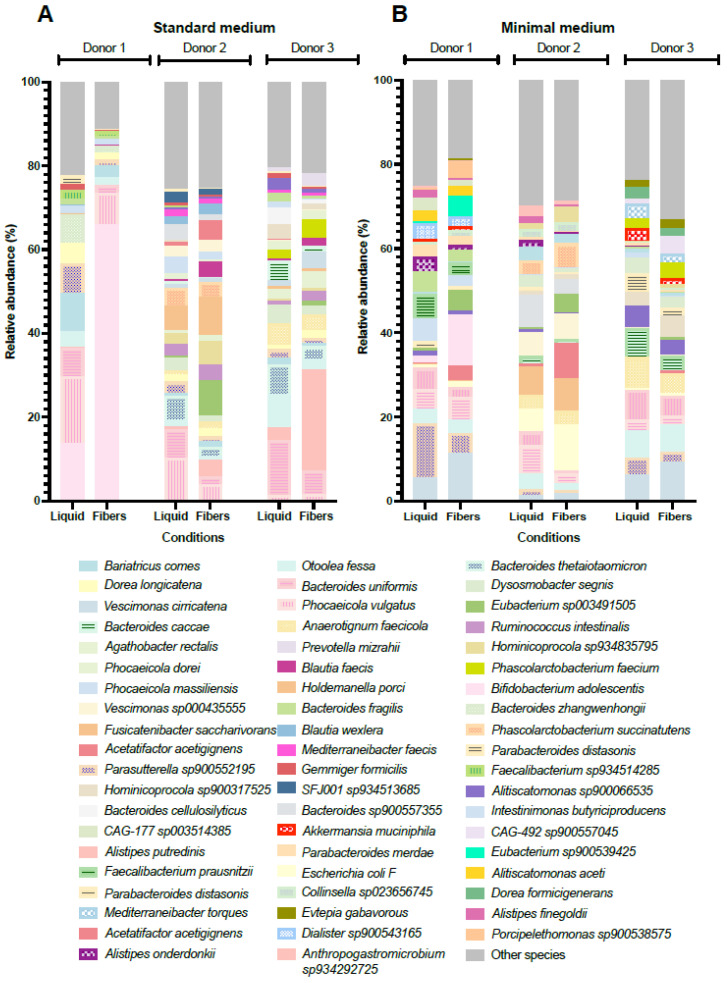
Relative abundance profiles of the 40 most abundant microbial species detected in fiber-associated and planktonic (liquid) fractions under different fermentation conditions. (**A**) Standard Medium; (**B**) Minimal Medium.

Differences in microbial community composition between fiber-associated and planktonic (liquid-phase) fractions were evaluated after 48 h of fermentation (Figure 9).

- Standard medium:

In SM, differences in microbial community composition between fiber-associated and planktonic fractions were observed for donor 1. *Bifidobacterium adolescentis* represented a larger proportion of the microbial profile recovered from the fiber fraction (65%) compared with the liquid phase (13%), suggesting a preferential representation of this taxon in the fiber-associated fraction under these conditions.

For donors 2 and 3, no single dominant species was observed on the fibers, consistent with the high alpha diversity reported above. However, some taxa displayed higher relative abundances in the fiber-associated fraction compared with the liquid fraction, such as *Anthropogastromicrobium* sp., particularly in donor 3 (up to 24%). Overall, differences between fiber-associated and planktonic communities remained limited in SM, likely due to the presence of readily fermentable carbohydrates in the medium.

- Minimal medium:

In MM, the microbial profiles associated with the fiber fraction remained diverse across all donors, with no single species dominating the fiber fraction. Nevertheless, several taxa displayed higher relative abundances in the fiber-associated fraction compared with the liquid phase.

For donor 1, *Bifidobacterium adolescentis* represented a higher proportion of the fiber-associated fraction (12%), along with *Vescimonas cirricatena* (11.5%). A similar increase in relative abundance of *Vescimonas cirricatena* was observed in donor 3 (9.3%). In donor 2, the fiber-associated fraction showed higher relative abundances of *Escherichia coli F* (10.8%) and *Acetatifactor acetigignens* (8.6%).

Despite these differences in relative abundance profiles, no consistent dominant taxon emerged across donors, suggesting that multiple microbial populations may interact with the fiber fraction. These results suggested that bamboo fibers may influence the spatial distribution of microbial communities under nutrient-limited conditions, without strongly selecting for a single dominant taxon.

Overall, the comparison between fiber-attached and planktonic microbiota highlighted that bamboo fibers can act as a niche for microbial colonization, particularly in minimal medium, while maintaining a diverse community structure.

## 4. Discussion

This study investigated the gastrointestinal digestibility and colonic fermentability of bamboo fibers using in vitro enzymatic digestion and fecal microbiota fermentation. The main findings indicate that bamboo fibers were resistant to enzymatic digestion in the upper gastrointestinal tract but supported fermentation activity of human gut microbiota under specific experimental conditions.

FTIR and SEM analyses confirmed that bamboo fibers retained their structural integrity after simulated salivary, gastric, and intestinal digestion, in contrast to wheat starch, which was effectively hydrolyzed. Reducing sugar assays further supported these observations, showing starch breakdown in the oral and intestinal phases, whereas bamboo remained intact. These results were consistent with the high contents of cellulose, hemicellulose, and lignin in bamboo fibers, which form a recalcitrant matrix resistant to enzymatic hydrolysis [11,22,23].

In vitro fermentation demonstrated that bamboo fibers supported fermentation-associated microbial activity in fecal microbiota from all three donors. In the presence of bamboo fibers, the concordance between increased gas production, enhanced acidification, and elevated SCFA concentrations in minimal medium supported the occurrence of microbial fermentation activity associated with bamboo supplementation. In this context, bamboo fibers represented the main available complex carbohydrate source, which was associated with increased levels of major SCFAs, including acetate, propionate, and butyrate. In standard medium containing readily fermentable carbohydrates, these effects were minimal, indicating that microbial communities preferentially metabolize simpler sugars over complex polysaccharides. This highlights that bamboo fiber fermentability was strongly dependent on nutrient availability and donor microbiota composition and may be underestimated in the presence of more accessible carbon sources, consistent with previous observations on hierarchical utilization of dietary fibers [22,38,39]. The structural recalcitrance of bamboo fibers, as previously described, likely limits microbial accessibility and slows down fiber transformation kinetics, which may explain why fermentation of bamboo fibers was primarily observed under substrate-limited conditions.

Our results are broadly consistent with previous in vitro studies showing that bamboo-derived substrates are fermentable and lead to SCFA production and acidification [7,8]. In Ge et al. [7], bamboo fiber supplementation increased acetate and propionate concentrations by +13.7% (2.16 vs. 1.90 g/L) and +24.3% (0.46 vs. 0.37 g/L), respectively. In the present study, larger relative increases were observed under minimal-medium conditions, where acetate increased by +63.6% (5.4 vs. 3.3 g/L) and propionate by +94.7% (1.48 vs. 0.76 g/L). However, these values should not be interpreted as evidence of higher intrinsic fermentability compared with previous studies, as the experimental contexts differ substantially. While Ge et al. [7] evaluated bamboo supplementation in nutrient-rich medium, the present study assessed bamboo under carbohydrate-limited conditions. The larger relative changes observed here likely reflect the absence of competing fermentable substrates rather than an intrinsically higher fermentative capacity of bamboo fibers. A notable difference is that in our study, the strongest fermentation response associated with bamboo supplementation was observed when alternative carbon sources were absent. This may partly reflect differences in microbial community composition, but other factors including donor age, habitual diet, inoculum variability, and experimental conditions should also be considered [40,41]. Additionally, our study provides new mechanistic insights through SEM observations and analysis of fiber-attached versus planktonic microbiota, revealing structural alterations of bamboo fibers and donor- and condition-dependent associations between microbial communities and bamboo fibers not previously reported.

Metagenomic analyses revealed donor- and condition-dependent shifts in the relative abundance of several taxa associated with bamboo supplementation, including members of *Bacteroides* (*B. thetaiotaomicron, B. uniformis, B. caccae*) and *Intestinimonas*. In contrast to Ge et al., who reported broad phylum-level shifts characterized by an increase in Firmicutes and decreases in Actinobacteria and Bacteroidota, our minimal-medium model revealed a more targeted compositional response at the genus level, involving specific members of Bacteroidota (*Bacteroides uniformis*, *B. thetaiotaomicron*, *B. caccae*), Firmicutes (*Intestinimonas*, *Faecalibacterium*), and Alistipes, without evidence of a global phylum-level restructuring. These compositional shifts should be interpreted cautiously, as changes in relative abundance do not necessarily indicate direct utilization of bamboo fibers but may also reflect indirect ecological interactions within the microbial community. While some taxa are known to utilize fiber-derived breakdown products, the observed changes in relative abundance may reflect cross-feeding interactions, where secondary fermenters may benefit from products generated by primary degraders. This highlights the ecological complexity of fiber fermentation and the limitations of inferring direct degraders from relative abundance alone [42,43]. Future integration of functional CAZyme analyses and transcriptomic approaches will be necessary to identify microorganisms actively involved in bamboo fiber utilization. SEM observations after 48 h of fermentation showed surface erosion and structural alterations of bamboo fibers, suggesting microbial-associated structural modification of bamboo fibers when considered together with metabolic indicators. In addition, the analysis of fiber-associated microbial fractions suggests that bamboo fibers may provide a physical niche for microbial association, although the mechanisms underlying these interactions remain to be elucidated.

Several limitations should be acknowledged. The limited number of donors (*n* = 3) does not allow population-level conclusions, and the observed responses should therefore be considered donor-specific observations requiring validation in larger cohorts. In addition, metagenomic data based on relative abundances provide evidence of community shifts, while SEM observations indicate structural modifications but neither approach directly demonstrates microbial growth or enzymatic degradation of bamboo fibers. Finally, although pH was readjusted twice daily to maintain microbial activity and avoid excessive acidification in this simplified batch fermentation model, this approach does not reproduce the continuous pH regulation occurring in dynamic colonic models such as SHIME^®^ or ARCOL [32,33]. Consequently, the accumulation of fermentation products and the resulting microbial community structure may differ from those occurring under physiological conditions. Future studies combining functional approaches, such as CAZyme profiling, absolute microbial quantification, and in vivo validation, will help further elucidate the mechanisms underlying bamboo fiber utilization. Furthermore, future continuous fermentation models with physiological pH evolution would improve the ecological relevance of the approach.

In conclusion, bamboo fibers resist enzymatic digestion but can support fermentation activity of human gut microbiota under carbohydrate-limited conditions. Fermentation is associated with increased acidification, gas production, and SCFA synthesis, together with donor- and condition-dependent shifts in microbial community composition. These findings extend previous observations obtained with bamboo-derived substrates and demonstrate that bamboo fibers have fermentative potential under carbohydrate-limited conditions using fecal microbiota from three donors. Further studies involving larger donor cohorts, continuous colonic fermentation models, CAZyme profiling, metabolomics, and in vivo validation will be required to better define the mechanisms underlying bamboo fiber utilization and transformation and their potential applications in functional food development.

## Data Availability

The original contributions presented in the study are included in the article, further inquiries can be directed to the corresponding author.
